# The inhibition of FGF receptor 1 activity mediates sorafenib antiproliferative effects in human malignant pleural mesothelioma tumor-initiating cells

**DOI:** 10.1186/s13287-017-0573-7

**Published:** 2017-05-25

**Authors:** Alessandra Pattarozzi, Elisa Carra, Roberto E. Favoni, Roberto Würth, Daniela Marubbi, Rosa Angela Filiberti, Luciano Mutti, Tullio Florio, Federica Barbieri, Antonio Daga

**Affiliations:** 1 Department of Internal Medicine (DiMI) and Centre of Excellence for Biomedical Research (CEBR), University of Genova, Viale Benedetto XV, 2, 16132 Genova, Italy; 2 Department of Experimental Medicine (DIMES), University of Genova, Via L.B. Alberti, 2, 16132 Genova, Italy; 3 IRCCS-AOU San Martino-IST, Largo R. Benzi, 10, 16132 Genova, Italy; 4 Biomedical Research Centre, University of Salford, The Crescent, Salford, Manchester, M5 4WT UK

**Keywords:** Pleural mesothelioma, Tumor-initiating cells, Sorafenib, Fibroblast growth factor, Apoptosis

## Abstract

**Background:**

Malignant pleural mesothelioma is an aggressive cancer, characterized by rapid progression and high mortality. Persistence of tumor-initiating cells (TICs, or cancer stem cells) after cytotoxic drug treatment is responsible for tumor relapse, and represents one of the main reasons for the poor prognosis of mesothelioma. In fact, identification of the molecules affecting TIC viability is still a significant challenge.

**Methods:**

TIC-enriched cultures were obtained from 10 human malignant pleural mesotheliomas and cultured in vitro. Three fully characterized tumorigenic cultures, named MM1, MM3, and MM4, were selected and used to assess antiproliferative effects of the multi-kinase inhibitor sorafenib. Cell viability was investigated by MTT assay, and cell cycle analysis as well as induction of apoptosis were determined by flow cytometry. Western blotting was performed to reveal the modulation of protein expression and the phosphorylation status of pathways associated with sorafenib treatment.

**Results:**

We analyzed the molecular mechanisms of the antiproliferative effects of sorafenib in mesothelioma TIC cultures. Sorafenib inhibited cell cycle progression in all cultures, but only in MM3 and MM4 cells was this effect associated with Mcl-1-dependent apoptosis. To investigate the mechanisms of sorafenib-mediated antiproliferative activity, TICs were treated with epidermal growth factor (EGF) or basic fibroblast growth factor (bFGF) causing, in MM3 and MM4 cells, MEK, ERK1/2, Akt, and STAT3 phosphorylation. These effects were abolished by sorafenib only in bFGF-treated cells, while a modest inhibition occurred after EGF stimulation, suggesting that sorafenib effects are mainly due to FGF receptor (FGFR) inhibition. Indeed, FGFR1 phosphorylation was inhibited by sorafenib.

Moreover, in MM1 cells, which release high levels of bFGF and showed autocrine activation of FGFR1 and constitutive phosphorylation/activation of MEK-ERK1/2, sorafenib induced a more effective antiproliferative response, confirming that the main target of the drug is the inhibition of FGFR1 activity.

**Conclusions:**

These results suggest that, in malignant pleural mesothelioma TICs, bFGF signaling is the main target of the antiproliferative response of sorafenib, acting directly on the FGFR1 activation. Patients with constitutive FGFR1 activation via an autocrine loop may be more sensitive to sorafenib treatment and the analysis of this possibility warrants further clinical investigation.

**Electronic supplementary material:**

The online version of this article (doi:10.1186/s13287-017-0573-7) contains supplementary material, which is available to authorized users.

## Background

Malignant pleural mesothelioma (MPM) is an aggressive and heterogeneous cancer that, after transient response to first-line treatments, relapses rapidly [1]. MPM still has a poor prognosis, with a median survival of 9–17 months, only slightly improved by the introduction of folate inhibitor/cisplatin combination chemotherapy [2]. Ongoing clinical trials are expected to provide information about the efficacy of the association of standard treatment with new targeted molecules [3].

The evolving theory of cancer stem cells (CSCs) postulates that the capacity to drive tumor formation and growth resides in a subpopulation of tumor cells, namely CSCs or tumor-initiating cells (TICs) [4]. CSCs are able to self-renew, differentiate into heterogeneous nontumorigenic cancer cells forming the bulk of tumor mass, and develop tumors in murine models. These cells also possess intrinsic chemoresistance and radioresistance and act as a reservoir of cancer cells responsible for relapse after surgery, radiation, or chemotherapy [5]. CSCs, identified in many human solid cancers including MPM, are mechanistically responsible for treatment failure, tumor relapse, and metastasis development [5]. Thus, the identification of CSC-targeting drugs represents a translationally relevant approach to improve cancer therapy [6], in particular to overcome refractoriness to conventional anticancer agents.

Preclinical CSC-based models, suitable to study the mechanisms by which novel drugs target tumor cells, are much less developed in MPM than in other solid tumors [6]. Candidate MPM CSCs were mostly derived from established human cell lines [7–9] and only few studies described the isolation of CSCs from primary cultures of human MPM cells [10, 11].

Several preclinical studies reported significant results in MPM using tyrosine kinase inhibitors (TKIs), targeting overexpressed and/or autocrinally activated growth factor receptors [12–14]. Unfortunately, most of them failed to achieve the expected survival benefits when translated into the clinical setting [15], possibly because MPM growth is sustained by deregulation of several receptor tyrosine kinases (RTKs), and the inhibition of a single pathway is insufficient to give clinical benefits. Moreover, substantial evidence validates the cross-talk among RTKs, including epidermal growth factor (EGF), basic fibroblast growth factor (bFGF), vascular endothelial growth factor (VEGF), and platelet-derived growth factor (PDGF) receptors, and their aberrant signaling in cancer. Thus, the use of drug combinations or multi-targeted agents acting on several RTKs and/or cytosolic kinases along downstream pathways, offers a more promising approach for the treatment of MPM. Investigations on the molecular pathogenesis of MPM highlighted signal transduction dysregulation in key pathways connecting RTKs and MAPK cascade. Consistently, dual targeting of TKs and signaling pathways in MPM patients represents a promising treatment option [3].

In this article, we investigated the antiproliferative effect of sorafenib, an orally available multi-kinase inhibitor with potent activity against *w.t.* Raf kinases (CRAF and BRAF) and the V600E BRAF mutant, along the MAPK pathway, and cell surface RTKs (VEGFR-2 and VEGFR-3, PDGFR-β, c-KIT, RET, FLT-3, and, with slightly lower potency, FGFR1) [16]. Sorafenib is FDA-approved for the treatment of advanced renal cell carcinoma (RCC) [17], hepatocellular carcinoma (HCC) [18], and differentiated thyroid cancer (DTC) [19]. In preclinical studies, monotherapies or combination therapies with sorafenib are effective against several tumors, preferentially affecting CSC viability [20–23]. However, the role of Raf-dependent and Raf-independent signaling inhibition in the antitumor activity of sorafenib and the precise molecular mechanisms of its activity are still not fully characterized [24].

In this context, we explored the activity of sorafenib against human MPM cell cultures enriched in TICs, and the molecular mechanisms involved. We demonstrate that sorafenib exerts antiproliferative and proapoptotic effects, the latter being mediated by the downregulation of Mcl-1. Moreover, we show that sorafenib activity is mainly dependent on the inhibition of FGFR1 signaling rather than downstream kinases. We show that MPM TIC cultures secreting high levels of bFGF, which induce an autocrine/paracrine activation of FGFR1, were the most responsive to sorafenib. Thus, it is likely that a subset of MPM patients displaying higher FGFR1 activity could be more sensitive to sorafenib, highlighting that accurate patients’ selection may offer the best therapeutic approach.

## Methods

### Chemicals

Sorafenib (US Biological) and AZ628 and PD173074 (Sigma-Aldrich) were dissolved in DMSO at 10 mM concentration and stored at –20 °C. Drugs were diluted with culture medium to the experimental concentrations, with a maximum 0.1% (v/v) DMSO final concentration. Corresponding vehicle concentrations were added to control samples.

### Cell cultures

Ten cultures (MM1–MM10) were obtained from postsurgical specimens of human MPMs (IRCCS-AOU San Martino-IST, Genova, Italy) upon approval of the institutional bioethics board and informed written consent from the patients [10]. Cells were cultured in DMEM/F12 (Gibco) supplemented with 2 mM l-glutamine (Gibco), bFGF (10 ng/ml) and EGF (20 ng/m) (Peprotech), 15 μg/ml insulin, and 2 μg/ml heparin (Sigma-Aldrich). However, only MM1–MM4 cells showed tumorigenic activity in vivo and were routinely xenografted in immunodeficient mice to ensure the maintenance of stemness. Cells recovered from tumor xenografts grow as tumorspheres, but prior to performing in-vitro experiments were allowed to attach in plastic flask by culturing them for short periods in medium containing 4% FBS (EuroClone). To avoid phenotypical and biological alterations caused by the culture conditions, all experiments were performed on cells after very low number of in-vitro passages. Phase-contrast images of cultures were acquired by a Nikon TE300 microscope.

### Mice xenografts

NOD-SCID mice (Charles River, Milan, Italy) aged 4–6 weeks were used to test their ability to grow in vivo. All animal procedures were carried out under project license in compliance with guidelines approved by the Ethical Committee for animal use in cancer research at IRCCS-AOU San Martino-IST (Genova, Italy) and the Italian Ministry of Health (n° 327, Dl.vo 116/92 and 412).

Xenografts were established by pseudo-orthotopic i.p. inoculation of MM1, MM3, and MM4 cells derived from cultured spheres. Mice were monitored for disease symptoms and sacrificed by CO_2_ asphyxiation when they showed weight loss or any sign of suffering. Excised tumors were divided into two parts: one part was cut into small fragments, and a cell suspension was collected for in-vitro testing and cultivated as already described. The second part was cryopreserved and stored for immunohistochemical analysis.

### Immunohistochemistry

Tumor xenograft cryo-sections were fixed in 4% PFA, treated with 0.4% pepsin in 0.2 N HCl, 3% H_2_O_2_–PBS, and then permeabilized and assayed for WT-1 (Dako) expression with anti-mouse EnVision-HRP (Dako). Calretinin antibody (Dako) was used on sections fixed in 4% PFA, followed by heat-induced antigen retrieval and treatment with 0.3% H_2_O_2_–PBS and permeabilization. Detection was performed using streptavidin/horseradish peroxidase (Dako). Mesothelin staining was assessed using a secondary antibody conjugated with a green fluorescent dye. For D2-40 staining (Dako), heat antigen retrieval was performed using Target Retrieval Solution S1700 (Dako) and permeabilization in PBS 10% NGS 0.3% Triton X-100. Mouse IgG and the omission of primary antibody were used as negative controls [25]. Digital images were captured by a Nikon Eclipse microscope (Nikon Europe).

### FACS analysis

MM1, MM3, and MM4 cells, stained with specific surface marker antibodies, conjugated with FITC or PE (BD Biosciences) or corresponding isotype antibodies, were analyzed using a FACScalibur (BD Biosciences), equipped with BD CellQuest Pro software, as reported [26].

### Cytotoxicity assay

Cells (3000 cells/well) were allowed to attach overnight as already described. Sorafenib, or vehicle, was added and cell viability was assessed after 24–72 h of treatment using the MTT assay [27]. IC_50_ values were calculated using nonlinear regression curve fit analysis with Graph Pad Prism 5.02 [28]. Each treatment was analyzed in quadruplicate, and the experiments were repeated at least three times.

### Apoptosis detection

Cells were treated with vehicle or sorafenib (at the concentration corresponding to the IC_50_ and 2 × IC_50_ values) and stained with Annexin V-FITC and propidium iodide (PI) (Apoptosis Detection kit; eBioscience). Samples were analyzed by FACS. The percentage of cell death was obtained by summing-up the percentages of early and late apoptosis [29].

### Cell cycle analysis

Control and treated cells were fixed with ethanol at 4 °C for 1 h, and resuspended in staining solution (PBS, 20 mg/ml RNAse A, 50 μg/ml PI, 0.5% Triton X-100; Sigma-Aldrich). The DNA content was quantified by FACS and the cell cycle profile analyzed by Listmode data using ModFit™ LT software (Verity Software House) [30]. At least 10,000 events per experimental point were collected, gating single nuclei and excluding cell aggregates. Data are the average of three independent experiments.

### BrdU incorporation assay

Cells were seeded into 96-well plates and treated with sorafenib for 48 h. DNA synthesis was evaluated by a colorimetric immunoassay for the measurement of 5-bromo-2′-deoxyuridine (BrdU) incorporation in proliferating cells (Cell proliferation ELISA; Roche), as reported [31]. Results are expressed as percentages of the value of untreated cells.

### Western blotting

To reduce basal phosphorylation, cells were cultured for 24 h in growth factor/serum-free medium. Starved cells were pretreated with vehicle or sorafenib for different times (15–180 min), and then either EGF (20 ng/ml) or bFGF (10 ng/ml) was added for 10 min. Total protein content was measured from whole cell lysates as described previously [32]. Equal amounts of proteins were size-fractionated by SDS-PAGE, and transferred onto PVDF membrane (Bio-Rad Laboratories). Blots were probed with the following antibodies: phospho-ERK1/2 (Thr201-Tyr204, #9101), phospho-MEK (Ser217/221, #9121), phospho-Akt (Ser473, #9271) phospho-STAT3 (Ser-727, #9134), Mcl-1 (#5453), phospho-FGFR (Tyr653/654, #3476), and FGFR1 (#9740) from Cell Signaling and α-tubulin (#T5168; Sigma-Aldrich). Secondary mouse and rabbit HRP-linked antibodies were from GE Healthcare. Detection of immunocomplexes was performed using Clarity ECL Western Blot (Bio-Rad Laboratories). All experiments were repeated at least three times. Densitometric analysis of bands was performed using ChemiDoc imaging system (Bio-Rad Laboratories).

### bFGF release assay

Triplicate cell samples were seeded to obtain a semiconfluent monolayer in Petri dishes and cultured in growth factor/serum-free medium for 24 h. Thereafter, the medium was collected and the levels of bFGF were measured using the Quantikine ELISA (R&D Systems) according to the manufacturer’s instructions.

### Statistical analysis

All experiments were repeated at least three times. Data from quantitative experiments are expressed as mean ± SEM. Statistical analyses were performed by two-tailed *t* test or one-way ANOVA with Tukey’s and Dunnett’s post test, using GraphPad Prism 5.02. Statistical significance was established at *p* < 0.05.

## Results

### Isolation and in-vitro and in-vivo expansion of TIC-enriched cultures from human MPMs

**Fig. 1:**
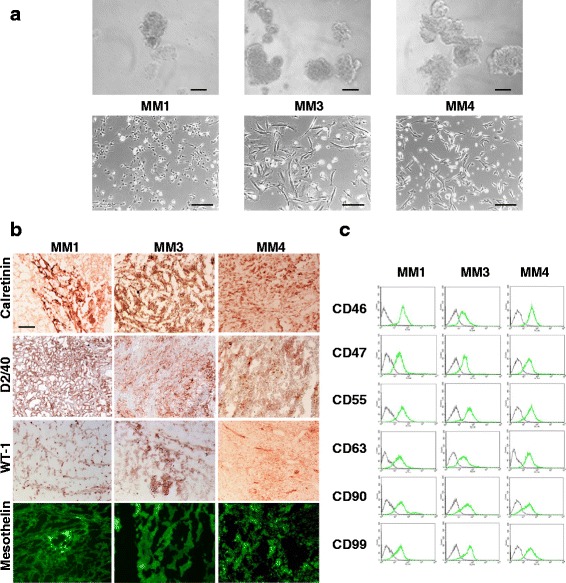
Characterization of TIC cultures derived from human MPMs. **a** Morphology of MM1, MM3, and MM4 TICs maintained in stem cell-permissive conditions: *upper panels*, sphere-forming cells derived from xenografted tumors in mice and grown as nonadherent clusters (*scale bar* 20 μm); *lower panels*, representative images of monolayers obtained by spheres (*scale bar* 100 μm). **b** Histological sections of tumors recovered from mice xenografted with MM1, MM3, and MM4 cells stained with the indicated antibodies. Immunoreactivity was detected by immunohistochemistry and immunofluorescence. Representative images are reported (*scale bar* 200 μM). **c** MM1, MM3, and MM4 cells, recovered from primary xenografts, analyzed by FACS for the expression of the indicated molecules. *Green*, cells stained with appropriate PE or FITC-conjugated antibodies; *gray*, cells incubated with PE or FITC-conjugated isotype-specific control antibody (Color figure online)

A series of primary MPM cell cultures (MM1–MM10) were isolated from human MPM specimens on the basis of their ability to grow in vitro under low-serum conditions. Detailed isolation procedures have been described elsewhere [10]. All cultures were characterized for tumor surface or stem-like marker expression, namely CD46, CD47, CD55, CD56, CD63, CD90, and CD99 (Additional file 1: Table S1). Among the analyzed MPM long-term cultures, four showed tumorigenic properties when xenografted in NOD-SCID mice, suggesting that they were enriched in the TIC subpopulation [10]; three of these cultures (MM1, MM3, and MM4), all derived from epithelioid MPMs, were selected to study the antiproliferative effects of sorafenib due to their sustained proliferation rate in vitro. In particular, MM1, MM3, and MM4 cultures used in this study derive from cells recovered from peritoneal tumors developed after injection in mice, a procedure that favors the enrichment in TICs. All the three TIC cultures develop tumor spheres when grown under anchorage-independent culture conditions (Fig. 1a, upper panels), but, to allow a better cellular and biochemical evaluation, proliferation and biochemical experiments were performed on short-term monolayer cultures obtained after disaggregation of the spheres (Fig. 1a, lower panels). The tumorigenic ability of MM1, MM3, and MM4 cells derived from spheroids was checked routinely and, in order to confirm their mesothelial phenotype and the persistence of TIC subpopulations, cryosections of pseudo-orthotopic xenografts were analyzed for the expression of widely recognized mesothelial markers (mesothelin, calretinin, D2-40, and WT-1 [33, 34]) by immunohistochemistry or immunohistofluorescence. Tumors generated from all MPM cultures were strongly positive for calretinin, confirming that the in*-*vitro growth did not modify the epithelioid subtype phenotype of the tumor of origin, while the expression of D2-40, WT-1, and mesothelin supported their mesothelial origin (Fig. 1b). Similarly, by FACS analysis, we show that in-vivo propagated cells retain the original phenotype as far as surface markers (i.e., high expression of CD46, CD47, CD55, CD56, CD63, CD90, and CD99) (Fig. 1c). The expression of stemness markers was also assessed by RT-PCR and immunofluorescence, showing the constant expression of BMI-1 but failing to detect the expression of SOX2, Nanog, Oct-4, or CD133, further confirming that these TIC-enriched cultures retain the original phenotype profile (data not shown). These findings confirm the enrichment in MPM TICs in the three selected cultures, thus representing a reliable experimental model to perform the study.

### Sorafenib inhibits cell proliferation in human MPM TIC-enriched cultures, in a concentration and time-dependent manner

**Fig. 2:**
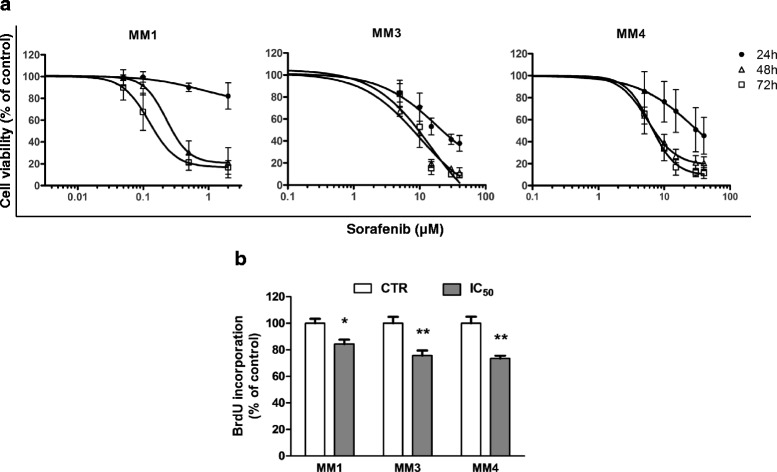
Sorafenib impairs cell viability and proliferation in MPM primary cultures enriched in TICs. **a** Time and dose-dependent effects of sorafenib on cell survival assessed by MTT assays, in MM1, MM3, and MM4 cells treated with increasing amounts of sorafenib for 24, 48, and 72 h. Each point represents quadruplicate replicates from at least three independent experiments. Data presented as the percentage of viable cells compared with control (vehicle-treated) cells, expressed as mean ± SEM. **b** Sorafenib impairs DNA synthesis in MPM TICs. Detection of proliferating cells was evaluated by BrdU DNA incorporation in MM1, MM3, and MM4 cells cultured for 48 h in the presence and the absence of sorafenib (administered at the respective IC_50_ values). Data expressed as mean % ± SD. *p <0.05, **p <0.01 vs respective controls. *CTR* control, *BrdU* 5-bromo-2′-deoxyuridine

In-vitro antiproliferative effect of sorafenib on TIC-enriched cultures was evaluated using increasing drug concentrations (up to 40 μM) and analyzing cell viability by MTT assay after 24–72 h (Fig. 2a). Sorafenib caused a concentration-dependent reduction of cell viability, more pronounced with increasing treatment time but, in MM3 and MM4 cells, already evident after 24 h.

IC_50_ values varied among cultures: at 48 h, MM1 cells were the most responsive to sorafenib (IC_50_ = 0.26 μM) whereas MM3 and MM4 cells displayed IC_50_ values of 6.40 and 5.97 μM, respectively.

Similar results were obtained in BrdU incorporation experiments, showing that sorafenib, used at concentrations corresponding to the respective calculated IC_50_, significantly decreased DNA synthesis in all cultures (Fig. 2b).

### Sorafenib induces cell cycle arrest in G0/G1 phase in MPM TICs

**Fig. 3:**
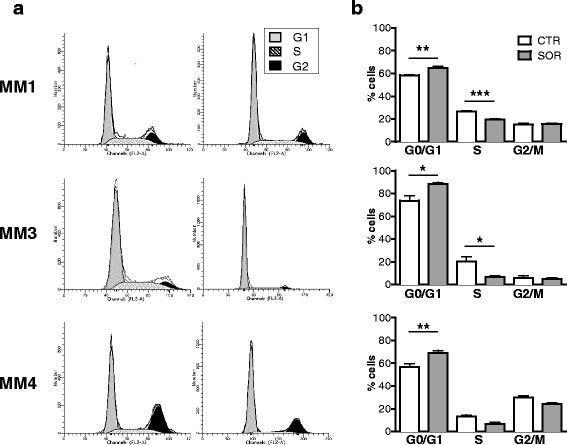
Sorafenib causes MPM TIC cell cycle arrest in the G0/G1 phase. **a** Representative cell cycle profiles of MM1, MM3, and MM4 cells treated with IC_50_ sorafenib or vehicle for 48 h before harvest, determined by staining with PI for DNA content analysis and counting on a FACScalibur. **b** Acquired FACS data were analyzed by ModFit LT software and the cell cycle phase distribution determined. Data correspond to mean ± SD of three independent experiments. *p <0.05, **p <0.01, ***p <0.001 vs respective controls. *CTR* control, *SOR* sorafenib

To further analyze the mechanisms mediating sorafenib antiproliferative activity, cell cycle analysis was carried out by FACS on PI-stained cells, treated for 48 h with concentrations corresponding to the IC_50_ (Fig. 3a). Sorafenib significantly increased the percentage of cells in the G1 phase in MM1 (from 58.42% ± 0.89 to 64.85 ± 2.74%, *p* = 0.037), MM3 (from 73.71 ± 8.78 to 88.34 ± 2.66%, *p* = 0.025), and MM4 (from 56.77 ± 5.68 to 69.05 ± 4.39%, *p* = 0.02) cultures (Fig. 3b). This effect was paralleled by a significant decrease of the percentage of cells in the S phase (MM1, from 26.48 ± 1.58 to 19.41 ± 1.44%, *p* = 0.003; MM3, from 20.31 ± 8.4 to 6.69 ± 2.37%, *p* = 0.049; MM4, from 13.41 ± 2.33 to 6.71 ± 2.66%, *p* = 0.009) (Fig. 3b). These data confirmed the BrdU incorporation results and suggest that sorafenib inhibits MPM TIC proliferation, impairing the G1/S transition.

### Sorafenib induces proapoptotic effects in MPM TICs via Mcl-1 downregulation

**Fig. 4:**
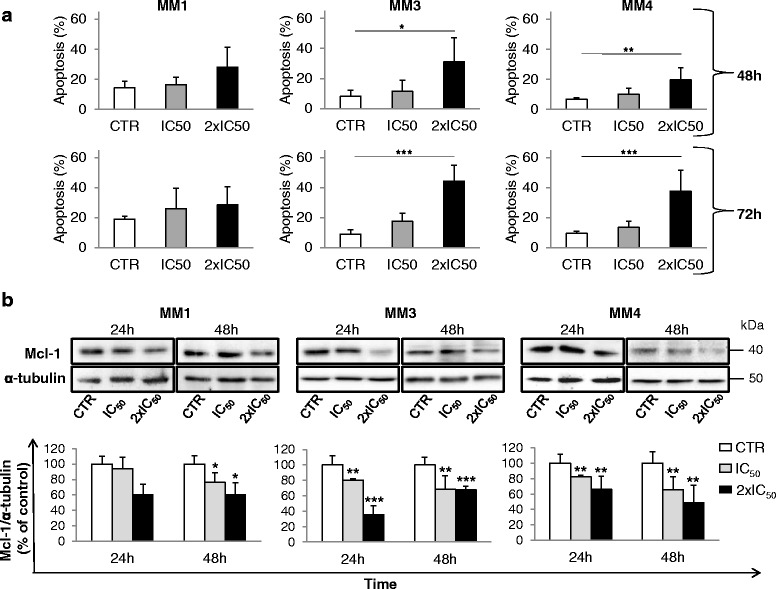
Sorafenib induces apoptosis and downregulates Mcl-1 expression in MPM TICs. **a** Amount of cell death (early and late apoptosis) in TIC cultures after IC_50_ and 2 × IC_50_ sorafenib exposure for 48 and 72 h. Data are mean ± SD of the percentages of Annexin V-positive cells (*n* = 3). Significant differences were detected between vehicle-treated and sorafenib-treated cancer cells. **b** Western blot analysis for Mcl-1 expression in cells treated with IC_50_ and 2 × IC_50_ sorafenib or control (vehicle-treated) for 24 and 48 h. Lysates were probed by western blot assay for Mcl-1 (*upper panels*). Blots were stripped and reprobed with anti-α-tubulin antibody to normalize for differences in protein loading. Immunoblotting was performed three times using independently prepared cell lysates. Data presented as mean ± SEM from different experiments (*lower panels*). Statistical significance in all panels of this figure was determined. *p <0.05, **p <0.01, ***p <0.001 vs respective controls. *CTR *control.

The contribution of apoptosis to the antiproliferative effects of sorafenib in the TIC cultures was analyzed after 48 and 72 h of treatment. Forty-eight hours of treatment with sorafenib, used at concentrations corresponding to the calculated IC_50_ (i.e., 0.26, 6.40, and 5.97 μM for MM1, MM2, and MM3, respectively) and 2 × IC_50_ (i.e*.*, 0.5, 13, and 12 μM, respectively) values, in MM3 and MM4 cultures induced a statistically significant increase in the number of apoptotic cells compared with vehicle-treated controls that became highly significant after 72 h (MM3, from 8.98 ± 2.8 to 17.71 ± 5.1% and 44.34 ± 10.6%; MM4, from 9.41 ± 1.6 to 13.6 ± 4.0% and 35.57 ± 13.8%) (Fig. 4a). Conversely, sorafenib-induced apoptosis did not reach statistical significance in the highly sensitive MM1 cells (Fig. 4a). We repeated these experiments using the same concentration of sorafenib (5 μM) in all cultures, to verify whether a higher sorafenib concentration is able induce a proapoptotic effect in MM1 cells, the IC_50_ value of these cells being 30-fold lower than MM3 and MM4 cells. However, the high sensitivity of MM1 cells to sorafenib prevented this evaluation, 5 μM being an excessively toxic concentration that resulted in necrosis of almost all of the cells (data not shown).

Proapoptotic effects of sorafenib in MPM TICs were associated with a strong downregulation of the anti-apoptotic protein Mcl-1, evaluated after 24 and 48 h of treatment (Fig. 4b). However, different levels of Mcl-1 inhibition across the cultures were observed, accordingly to the different ability of sorafenib to induce apoptosis. MM1 cells, which did not show statistically significant proapoptotic effects at the sorafenib concentrations used, exhibited a significant reduction of Mcl-1 only after 48 h (–24% and –39% at concentrations corresponding to IC_50_ and 2 × IC_50_, respectively; *p* < 0.05). MM3 and MM4 cells, showing a robust apoptotic response to sorafenib, displayed a highly significant reduction of Mcl-1 expression already after 24 h of treatment (–20% and –75% in MM3 and –18% and –34% in MM4, at IC_50_ and 2 × IC_50_, respectively), an effect that persisted after 48 h (Fig. 4b). Collectively, these data suggest that sorafenib triggers apoptosis in MPM TICs by impairing Mcl-1 expression.

### Sorafenib reduces EGFR signaling in MPM TICs

**Fig. 5:**
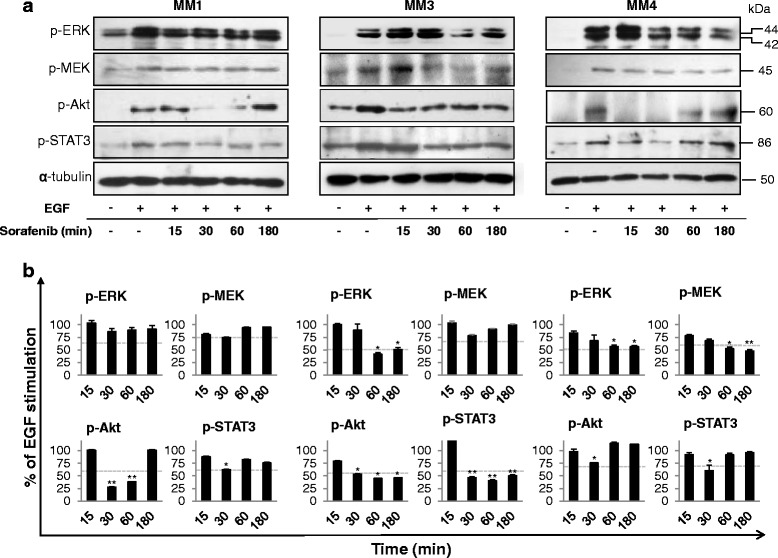
Effects of sorafenib treatment on intracellular signaling in EGF-stimulated MPM TICs. **a** Western blot analysis using antibodies specific to the phosphorylated proteins in MM1, MM3, and MM4 cells treated with IC_50_ sorafenib and stimulated with 20 ng/ml EGF for the indicated time periods (15–180 min). Representative immunoblots of three independent experiments are reported. **b** Images from independent experiments were quantified by band densitometric analysis and the resulting relative mean ratio of each protein/α-tubulin is shown. Values expressed as a percentage of the value obtained for the EGF-stimulated cells in the same blot. Results are mean ± SEM of three experiments. *p <0.05, **p <0.01. *Dotted lines*, basal (untreated) levels. *EGF* epidermal growth factor

Sorafenib impairs tumor growth by inhibiting multiple kinases involved in cancer cell survival and proliferation. Among them, it was reported that sorafenib mainly acts by inhibiting the MAPK cascade, via the direct inhibition of Raf kinase. To delve deeper into the intracellular effectors mediating antiproliferative and proapoptotic effects of sorafenib in MPM TICs, we analyzed its ability to modulate ERK1/2, Akt, and STAT3 signaling pathways by western blotting (Fig. 5a). To activate these pathways which represent the main effectors of mitogens in MPM cells, we treated TICs with EGF or bFGF, the growth factors commonly used to sustain in-vitro proliferation of TICs [35]. Time-course experiments were performed in MM cultures previously deprived of growth factors for 24 h, treated with vehicle or sorafenib at the IC_50_ values for each TIC culture for 15–180 min, and then stimulated with EGF (20 ng/ml), for 10 min. EGF treatment induced MEK and ERK1/2 phosphorylation in all MM TIC cultures (range +30/+60% over the basal); sorafenib pretreatment reduced, but did not abolish, the activation of these kinases (Fig. 5b). Sorafenib effects were time dependent, and variable among the different cultures. Maximal inhibition of ERK1/2 phosphorylation occurred after 60 min (–58%) in MM3 cells, while in MM4 cells the reduction of phospho-ERK1/2 (–43%) and phospho-MEK (–52%) was observed after 180 min (Fig. 5b). Interestingly, in MM1 cells a constitutive activation of MEK and ERK1/2 was observed that was further increased by EGF treatment (about +40%) but not reduced by sorafenib (Fig. 5b).

Because Akt provides cells with a survival advantage in the presence of apoptotic stimuli, the ability of sorafenib to affect Akt signal transduction was evaluated in EGF-treated cultures (Fig. 5a). EGF led to a significant activation of Akt in all TIC cultures, and sorafenib transiently reduced phospho-Akt levels in MM1 and MM4 cells (after 30–60 min ≈ 25–70% inhibition was detected, but this effect vanished after 180 min) (Fig. 5b). In MM3 cells, Akt phosphorylation also observed in growth factor-deprived control cells, was significantly increased by EGF treatment, and was brought back to basal by sorafenib over the time-course analysis (Fig. 5b). Therefore, EGF-mediated MAPK signaling remained partly active after sorafenib treatment in MM3 and MM4 cells, and was not affected in MM1 cells. Moreover, Akt phosphorylation induced by EGF quickly recovered from sorafenib inhibitory effects, suggesting compensatory activation of the phosphatidylinositol-3-kinase (PI3K) pathway.

To further identify intracellular mediators underlying the antiproliferative activity of sorafenib, we evaluated the activation of the signal transducer and activator of transcription 3 (STAT3) that mediates transcriptional regulation of genes involved in tumor cell survival in response to growth factors (Fig. 5a). Exposure to sorafenib transiently reduced EGF-dependent STAT3 phosphorylation in MM1 (maximum –37% after 30 min) and MM4 (maximum –40% after 30 min) cells, whereas in MM3 cells a sustained dephosphorylation starting 30 min after treatment (–55%) and lasting for the entire experimental period was observed (Fig. 5b).

Taken together, these data demonstrate that, besides individual differences among MPM TICs, all three cultures studied show, in response to sorafenib, a noncomplete inhibition of ERK1/2, Akt, and STAT3 pathways activated by EGF. Moreover, it is worthy to point out that a rather modest effect was observed on MEK activation, the direct target of Raf.

### Sorafenib abolishes bFGF induced intracellular signaling

**Fig. 6:**
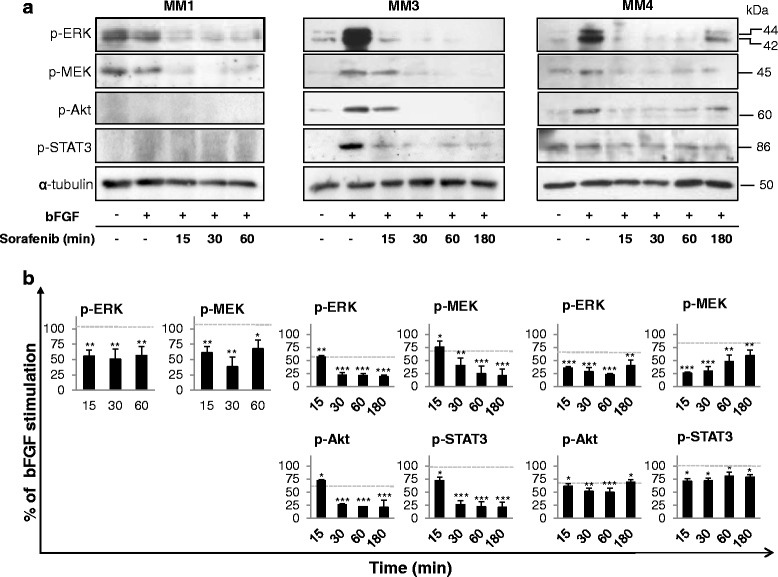
Sorafenib suppresses intracellular signaling in bFGF-stimulated MPM TICs. **a** Western blot analysis using antibodies specific to the phosphorylated proteins in MM1, MM3, and MM4 cells treated with IC_50_ sorafenib and stimulated with 20 ng/ml bFGF for the indicated time points (15–180 min). Representative immunoblots are reported. **b** Densitometric scanning of the immunoreactive bands followed by correction for α-tubulin loading control, from independent experiments. Values expressed as a percentage of the value obtained for the bFGF-stimulated cells in the same blot. Results are mean ± SEM of three experiments. *p <0.05, **p <0.01, ***p <0.001. *Dotted lines*, basal (untreated) levels. *bFGF* basic fibroblast growth factor

Signaling through the bFGF/FGFR1 plays a relevant role in MPM proliferation [36], and thus the effects of sorafenib on bFGF-induced intracellular pathways were investigated. We carried out parallel time-course (15–180 min) experiments treating growth factor-starved MPM TICs with sorafenib at the indicated IC_50_ for each culture, and then stimulated with 20 ng/ml bFGF for 10 min (Fig. 6a). In MM3 and MM4 cells, bFGF exposure caused a significant increase in MEK and ERK1/2 activation (+45% and +35%, respectively), while in MM1 cells a constitutive phosphorylation of MEK and ERK1/2 was observed and not further increased by bFGF (Fig. 6a, b). However, sorafenib abolished bFGF-dependent activation of both MEK and ERK1/2 in all TIC cultures (Fig. 6b), reaching a level of inhibition significantly greater than that observed after EGF treatment.

We also determined the effects of sorafenib on Akt and STAT3 phosphorylation induced by bFGF as downstream molecules involved in FGFR1-mediated proliferation. Sorafenib abrogated Akt and STAT3 bFGF-dependent activation (up to 80%) in MM3 cells, while the inhibition observed in MM4 was less evident, although statistically significant at all the experimental time points (Fig. 6b). Notably, in MM1 cells, no Akt and STAT3 phosphorylation was observed, and therefore, any inhibitory effect of the drug was detectable (Fig. 6a).

In their whole, these data show, in all the three cultures, a significant higher efficacy of sorafenib in blocking the MAPK pathway downstream to FGFR rather than to EGFR.

**Fig. 7:**
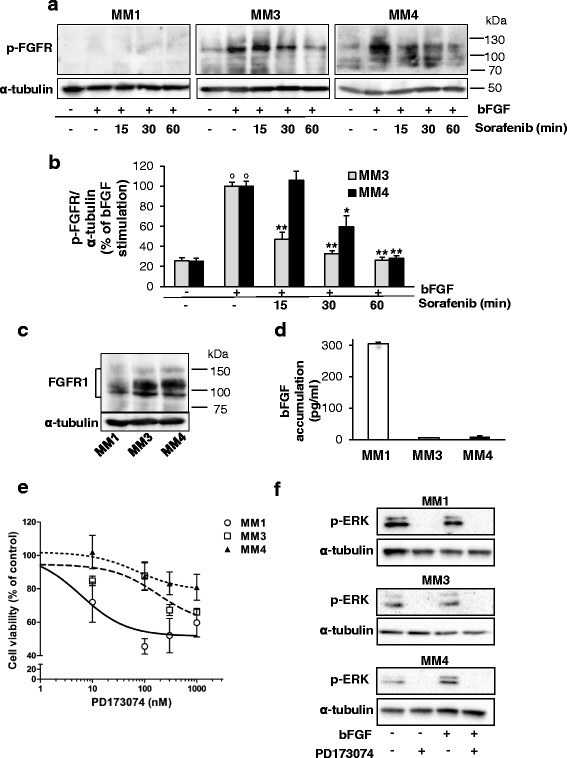
Sorafenib impairs FGFR1 activation in MPM TICs. **a** Western blot analysis using antibodies specific to phosphorylated FGFR in MM1, MM3, and MM4 cells stimulated with 20 ng/ml bFGF and treated with IC_50_ sorafenib for the indicated time points or vehicle-treated controls. Representative immunoblots are reported. **b** Data derived from densitometric analysis of at least three independent sets of experiments, expressed as mean ± SEM of the percentage values of the bFGF-induced FGFR phosphorylation (set as 100%). *p <0.05, **p <0.01 vs bFGF-stimulated cells; °*p* <0.05 vs vehicle-treated cells. **c** Western blot analysis using antibodies specific to total FGFR1 in MM1, MM3, and MM4 cells cultured in the absence of growth factors (EGF and bFGF). Blots were stripped and reprobed with anti-α-tubulin antibody to normalize for differences in protein loading. Representative immunoblots are reported (*n* = 3). **d** Quantitative evaluation of bFGF production in MPM cultures by ELISA. Data show the amount of bFGF released in 24 h by each TIC culture. **e** MPM TICs, serum-deprived for 24 h, were exposed to 0.001–1 μM PD173074 for a further 48 h and the effects analyzed by MTT assay. Values presented as percentage of the untreated control (100%) and represent mean ± SEM. **f** Western blot analysis of ERK1/2 phosphorylation in serum-starved conditions in the presence or absence of PD173074 (100 nM, 30 min) and/or bFGF (10 ng/ml, 10 min). Blots were reprobed for α-tubulin as protein loading control. *bFGF* basic fibroblast growth factor, *FGFR* FGF receptor

Thus, we measured the effect of sorafenib (at IC_50_, for up to 60 min) on FGFR1 phosphorylation status (Fig. 7a). We observed in MM3 and MM4 cells that the powerful activation of the receptor upon bFGF stimulation (4-fold increase as compared with vehicle-treated controls) was significantly and time-dependently decreased by sorafenib (about –75% after 60 min, Fig. 7b), suggesting that FGFR1 could represent a primary target by which sorafenib affects MPM TIC viability.

As expected from the reported kinase inhibition profile, sorafenib did not modify EGFR phosphorylation induced by EGF (data not shown).

Again MM1 cells showed a peculiar response: phospho-FGFR1 was undetectable by western blot assay, although total FGFR1 was present in these cells, even if at lower levels than in MM3 and MM4 cultures (Fig. 7c). Thus we hypothesized that the low expression of FGFR1 was dependent on a constant activation and a very rapid turnover of the receptor protein. This observation well-matched the basal activation of MEK and ERK1/2 we detected in MM1 cells (see Fig. 6a), indicative of a constitutive signaling from FGFR1. Indeed, an autocrine bFGF/FGFR activation loop controlling ERK1/2 signaling has been identified in many cancer histotypes [37]. To verify this hypothesis, we measured by ELISA, bFGF content in the conditioned medium of the three TIC-enriched cultures. MM1 cells released, in 24 h, more than 30–50-fold higher levels of bFGF (304.8 ± 5.4 pg/ml) than MM3 and MM4 cells (5.7 ± 0.30 and 9.3 ± 2.8 pg/ml, respectively) (Fig. 7d).

Thus, high amounts of bFGF produced by MM1 cells might lead to an autocrine constitutive activation of FGFR1 that consequently triggers both the enhanced basal FGFR1 signaling and the downregulation of its expression.

To further demonstrate the role of FGFR1 in MPM TIC proliferation and its targeting by sorafenib, the effect of the inhibition of FGFR1 activity by the small molecule inhibitor PD173074 [38] was investigated in growth factor-starved MM1, MM3, and MM4 cells, cultured for 48 h in the presence of concentrations of inhibitor ranging from 0.001 to 1 μM. A dose-dependent reduction of cell viability, measured by MTT assay, was indeed observed (Fig. 7e), indicating a constitutive activation of FGFR1 that in the absence of exogenous growth factor was likely dependent on autocrine activation by bFGF spontaneously released by the cells; moreover, dose–response curves revealed that MM1 cells display higher sensitivity to PD173074 (IC_50_ = 6.12 nM) in agreement with the higher amount of bFGF produced by these cells. Moreover, the inhibitory activity of PD173074 on MPM TICs was not additive with sorafenib effects (data not shown). These results confirm that FGFR1 activation, triggered by autocrine bFGF production, represents the major determinant for MPM TIC proliferation and the main target of the antiproliferative activity of sorafenib.

PD173074 treatment also prevented activation of FGFR1 downstream MAPK pathway signaling, abolishing phospho-ERK1/2 levels in all the three cultures. In particular, PD173074 was able to block both bFGF-induced and basal/constitutive ERK1/2 phosphorylation in MM1 cells, confirming that PD173074 prevents FGFR1-induced ERK1/2 activation (Fig. 7f).

**Fig. 8:**
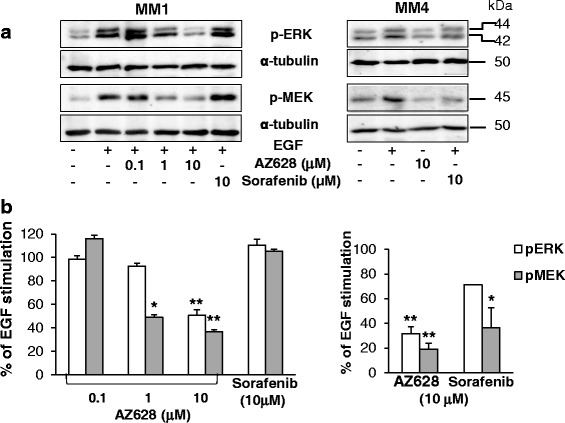
**a** Western blot analysis using antibodies specific to phospho-ERK1/2 and phospho-MEK in MM1 and MM4 cells stimulated with EGF in the presence or absence of AZ628 or sorafenib. α-tubulin was used to normalize protein loading. Representative immunoblots are reported. **b** Data derived from densitometric analysis of at least three independent experiments, using MM1 (*left*) and MM4 (*right*) cells. Data expressed as mean ± SEM of the percentage values of the EGF-induced ERK1/2 and MEK phosphorylation, set as 100%. *p <0.05, **p <0.01 vs EGF-stimulated cells. *EGF* endothelial growth factor

**Fig. 9:**
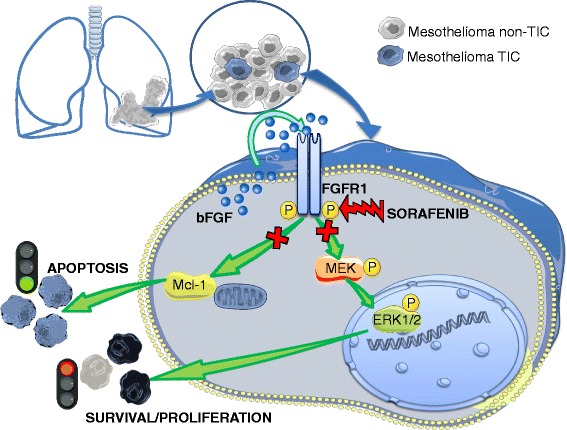
Diagram depicting the proposed mechanism of action of sorafenib in mesothelioma TICs. Sorafenib treatment causes the inhibition of MEK and ERK1/2 phosphorylation and the downregulation of Mcl-1 expression, leading to cell cycle arrest and activation of the apoptotic program. Sorafenib antitumor effects are mainly ascribed to a direct inhibition of FGFR1 activity rather than downstream effectors, such as Raf/Ras/MEK/ERK pathway. Thus TIC cultures autocrinally activating FGFR1 via the release of high amount of bFGF are likely to be more sensitive to the drug. *bFGF* basic fibroblast growth factor, *FGFR* FGF receptor, *TIC* tumor-initiating cell

Because antitumor activity of sorafenib is generally attributed to a direct inhibition of Raf, to verify the relative contribution of the inhibition of either FGFR1 or Raf kinase in sorafenib modulation of MEK/ERK signaling we used the pan-Raf kinase inhibitor AZ628, which also inhibits several tyrosine protein kinases like sorafenib. Western blot experiments in MM1 cells treated with AZ628 (0.1, 1, and 10 μM) showed a significant and concentration-dependent inhibition of MEK phosphorylation, the direct Raf target (around –60% at 1 and 10 μM) and its downstream substrate ERK1/2 (–49% at 10 μM) after EGF stimulation (Fig. 8a). Thus, in MM1 cells in which sorafenib fails to suppress the EGF-dependent activation of MEK and ERK1/2, AZ628 efficiently impaired the signaling cascade from Raf to MEK and, consequently, to ERK1/2. Moreover, as expected, the sustained EGF-dependent phosphorylation of MEK and ERK1/2 in the sorafenib-responsive MM4 culture was also significantly suppressed (–70 and –80%, respectively) by exposure to AZ628 (Fig. 8b). Overall these results suggest that bFGF-driven signaling plays a relevant role in the proliferation of MPM TICs and that cytotoxic and proapoptotic effects of sorafenib are mainly exerted by the direct inhibition of FGFR1, while the modulation of Raf activity seems less relevant (Fig. 9).

## Discussion

Despite the availability of multiple drug regimens, the survival rate of MPM patients is dramatically poor [3]. The identification of novel therapeutic approaches requires the development of preclinical in-vitro cellular models able to identify drugs with high probability to be efficacious in the clinic. Continuous cancer cell lines are commonly used as representative models of a given tumor histotype, but their translational value is often low. In solid and hematologic tumors, the presence of TIC/CSC subpopulations is at the basis of cellular heterogeneity. TICs have different phenotype and drug sensitivity than the bulk of tumor cells, more closely resembling cognate tumor profiles than commonly used cell lines, causing inter-tumor and intra-tumor variability not reproducible in established cell lines. In this respect, MPM TICs in vitro retain patient-specific traits and, due to the relatively short time required to obtain their enrichment in primary cultures, the genotypic and phenotypic modifications induced by in-vitro growth are minimized [39]. Hence, TIC cultures, although they pose several technical challenges, better reproduce the original tumor features than long-term established cell lines, in particular regarding drug responsiveness.

In this study, we used three individual MPM TIC cultures representing a suitable model to improve the value of studies in preclinical research. The number of patient-derived cultures we analyzed is not high due to the difficulties to obtain postsurgical samples and to establish primary mesothelioma stem cell-enriched cultures (only four of 10 cultures analyzed retained in-vivo tumorigenicity). Notwithstanding, we believe that this experimental model is highly representative of the MPM phenotype and its biological behavior, and is extremely useful to evaluate the effects of sorafenib on cell viability and the mechanisms involved.

Sorafenib is reported to exert antitumor effects via both the direct blockade of Raf in the ERK1/2 pathway and the inhibition of multiple RTKs. However, the precise pharmacological mechanisms responsible for its effects are still controversial and why sorafenib is not always effective in patients remains poorly understood [24]. Since sorafenib approval for RCC [17], HCC [18], and DTC [19], several clinical studies have investigated the effectiveness sorafenib in other tumors [40]. However, likely due to the molecular heterogeneity of these tumors, only few patients showed transient benefit from these trials, while disease progression occurred in most of the patients; therefore, determinants of clinical efficacy of sorafenib need confirmatory challenge. Analogously, after a phase I study which determined safety, pharmacokinetics, and efficacy of sorafenib in combination with doxorubicin in MPM patients [41], two phase II trials reported limited activity [42, 43]. The modest outcome of sorafenib as monotherapy in advanced MPM patients, although similar to that seen with other VEGFR TKIs [42], might be due to different factors, including the lack of MPM patient selection as a consequence of the absence of predictive markers for drug response. In particular, the identification of molecular mechanisms crucial for tumor cell proliferation affected by sorafenib and, possibly, ensuring drug synergism with cytotoxic drugs should be considered to identify potentially responsive patient subgroups. Overall, low number of patients entered the cited clinical trials, and the treatment of a molecularly undefined group of MPM patients concurs with disappointing in-vivo effectiveness of sorafenib, making their clinical significance uncertain. Moreover, because MPMs are not exclusively driven by gene mutation and amplification or RTK activation, and because intracellular pathways and autocrine/paracrine loops coexist and may interact with RTK functions, further molecular investigation and patient selection are warranted.

In this context, patient-derived TIC cultures may represent a novel preclinical model with higher translational predictivity, able to identify a subset of patients whose tumors show molecular characteristics predictive for positive clinical responses.

Previous in-vitro studies provided evidence that sorafenib targets TICs isolated from cell lines [44] and primary cultures [20] in other tumor types. Here we demonstrate that sorafenib also impairs MPM TIC viability, mainly via the inhibition of FGFR1 activity rather than downstream molecules.

Sorafenib effects occur in the nanomolar/low micromolar range in all the TIC cultures analyzed, showing similar sensitivity profiles and IC_50_ values to MPM cell lines [12, 45]. These data are in accordance with most studies reporting the cytotoxic effect in the 1–10 μM range of sorafenib in human cancer cell lines from different tumor types [46]. Efficacious sorafenib plasma concentrations achievable in patients are usually reported within the nanomolar range, although in some case concentrations within the micromolar range were reached [47], which are similar to the sorafenib concentrations effective in our cell model. Moreover, the concentrations at which sorafenib inhibits FGFR1 autocrine activation in MPM TICs are similar to those reported to affect the RAF/MEK/ERK pathway and activated RTK in human breast cancer cell lines [48]. Notwithstanding all these considerations, we have to acknowledge that the requirement of high sorafenib concentration to affect MPM TIC viability in vitro is still an issue when preclinical results have to be translated to the clinical setting, because kinase inhibitors are often associated with nonspecific dose-dependent toxic effects.

In this context, our results showing a nonhomogeneous sensitivity to the drug among TIC cultures isolated from different tumors suggest that distinctive molecular features determine the efficacy of the treatment. In particular, we show that TICs isolated from a human MPM (MM1) are highly responsive to sorafenib (IC_50_ = 260 nM) due to a constitutive autocrine activation of FGFR1, which we identified as the main molecular target in these cells, as discussed in the following. If this observation was to be confirmed in different patient-derived cultures, we can hypothesize that the identification of autocrine activation of this receptor could represent a starting point to identify subgroup of patients likely to be highly responsive to sorafenib.

As far as the mechanisms of action in MPM TICs, sorafenib treatment for 24–48 h induces G1 cell cycle arrest, as also reported in NSCLC [49] or thyroid cancer cell lines [50]. Interestingly, in our experimental model, cell death was mostly observed after 72 h of treatment, indicating that sorafenib inhibition of MPM TIC proliferation precedes the activation of the apoptotic process. Sorafenib-induced apoptosis was observed in MM3 and MM4 cultures, while apoptotic cell death, although present, was less relevant in MM1 cells using sorafenib at concentrations corresponding to the IC_50_. Cytotoxic activity of sorafenib in human cancer cells has been ascribed to downregulation of anti-apoptotic proteins as Mcl-1 [20, 51]; accordingly, we observed that sorafenib downregulates the expression of Mcl-1 in all the three MPM TIC cultures, an event contributing to the proapoptotic activity of this drug.

Ras/Raf/MEK/ERK and PI3K/Akt are the main signal transduction cascades involved in MPM development and progression, and in TIC proliferation and survival, while STAT3 contributes to maintain the transformed state and to promote metastasis [52]. To delve deeper into the molecular mechanisms mediating sorafenib activity, we tested its effects on MPM TICs in the presence of either EGF or bFGF. EGF is relevant for MPM pathogenesis and proliferation [12, 35, 52]. In MM3 and MM4 cells, sorafenib slightly attenuates EGF-dependent MEK, ERK1/2, and STAT3 phosphorylation, while Akt activation is transiently inhibited. Even if the extent of inhibition differs among MM1, MM3, and MM4 cells, sorafenib caused an incomplete reduction of EGF effects in all the cultures analyzed.

On their whole, these findings suggest that sorafenib-mediated inhibition of EGF-dependent MAPK signaling via Raf inhibition is not fully efficient in MPM TICs, and elevated levels of phosphorylated MEK and ERK1/2 are maintained even when Raf activity is theoretically completely blocked by sorafenib. To analyze the regulation of Raf/MEK/ERK signaling and to exclude that functional alterations and/or mutations in the MAPK pathway were responsible for the weak response to sorafenib, we used the pan-Raf inhibitor AZ628. AZ628 significantly inhibited EGF-dependent MEK and ERK1/2 phosphorylation in the sorafenib-responsive MM4 TICs. These results suggest that modulation of the MAPK pathway is readily achievable in MPM TICs and the low sorafenib efficacy is not due to intrinsic mechanisms of Raf activation. Thus, the direct inhibition of Raf seems to play a minor role in the antiproliferative activity of sorafenib in MPM TICs.

Because overexpression of alternative growth factors has been proposed as an escape mechanism from targeted therapies [53], we evaluated the activity of sorafenib in TICs stimulated with bFGF. Interestingly, sorafenib caused an almost complete abolishment of bFGF-induced phosphorylation of both MEK and ERK1/2. Akt and STAT3 activation by bFGF was also inhibited. The differential ability of sorafenib to inhibit MAPK cascade activated by bFGF and EGF is highly suggestive that sorafenib effects in MPM TICs have to be mainly ascribed to a direct inhibition of FGFR tyrosine kinase, rather than downstream effectors such as Raf. This evidence is further confirmed by the demonstration that sorafenib directly abrogates ligand-dependent FGFR1 (but not EGFR) phosphorylation.

Thus, we propose that FGFR1 is a major target of the antiproliferative activity of sorafenib in MPM TICs. The FGFR family, and FGFR1 in particular, is highly expressed in MPM cell lines [54], and represents an emerging therapeutic target for cancer treatment [55]. In several tumors, FGFR signaling drives tumorigenesis being activated by autocrine/paracrine loops [56]. For example, an aberrant autocrine bFGF circuit is a key component of downstream ERK1/2 activation and tumor aggressiveness in NSLC [57], breast cancer [58], head and neck squamous cell carcinoma [59], and mesothelioma [36] cells, and mediates resistance to RTK inhibitors [60]. Our data support that FGFR-driven signaling plays a relevant role in the biology of MPM TICs and that sorafenib cytotoxic and proapoptotic effects are mainly ascribed to inhibition of FGFR1 rather than a Raf-dependent mechanism. These data, quite unexpectedly, contrast to what is commonly observed in other tumor types in which sorafenib mainly acts through a direct inhibition of Raf kinase. Conversely, in the MPM TICs we analyzed, sorafenib was much more potent on FGFR1 than on RAF, although the latter kinase is active and functional, as shown by the efficient inhibition induced by the selective inhibitor AZ628. Importantly, sorafenib activity on receptor downstream signaling is commonly considered a key feature of this drug to interfere with different RTK activity. We show that, at least in MPM TICs, this does not occur and a direct effect on FGFR1 is mediating the antitumor efficacy of sorafenib. Actually, the mechanism of action of sorafenib in all three TIC cultures converges on FGFR1, this receptor being instrumental for the response to the drug of all the cultures analyzed (MM1, MM3, and MM4).

Indeed, the observation that sorafenib effects in all TICs are mainly mediated by the direct inhibition of FGFR1 activity can also explain the higher sensitivity of MM1 cells in comparison with MM3 and MM4 cells, as regards the antiproliferative activity of sorafenib (IC_50_: 0.26 μM in MM1 vs 6.40 and 5.97 μM in MM3 and MM4). In fact, MM1 cells display a constitutive activation of the MAPK pathway mainly dependent on the autocrine activation of FGFR1; in fact, untreated MM1 cells release high levels of bFGF, reaching concentrations about 30-fold higher than those of MM3 and MM4 cells. Thus, FGFR1 constitutive activity likely represents the main stimulus to MM1 cell proliferation and, consequently, being the key target of sorafenib effects, could determine the higher sensitivity of these cells. Notably, in MM1 cells, the release of large amounts of bFGF also caused an ultra-rapid turnover of FGFR1 activated form, likely sustained by the ligand autocrine loop, resulting in undetectable levels of receptor autophosphorylation even after bFGF exposure by western blot analysis, as described previously [61].

The FGFR1 activation/inactivation cycle is thus possibly boosted in cells with a strong bFGF production, although maintaining a sufficient amount of free receptor for binding, as reported [62], and also observed for chemokine receptors in human glioblastoma TICs [63].

Taken together, these data support that FGFR-driven signaling plays a relevant role in the proliferation and survival of MPM TICs and represents a common and determining factor mediating sorafenib antiproliferative affects, in all three cultures analyzed. Moreover, the level of bFGF secretion and autocrine activation of FGFR1 significantly impacts the entity of the antiproliferative response to the drug. Importantly, these data also highlight a pivotal Raf-independent mechanism underlying the cytotoxic and proapoptotic effects of sorafenib is evident in MPM TICs, in contrast with a direct inhibition of Raf proposed in different tumors. In particular, to the best of our knowledge, this is the first study demonstrating the relationship between bFGF production from tumor cells and the susceptibility to sorafenib of a TIC subpopulation. The translation of these results into a clinical setting could provide indications to select MPM patients likely responsive to treatment with sorafenib. The possibility of bFGF-driven proliferation and survival of tumor cells via autocrinally regulated pathways may be crucial to screen potentially responsive patients to targeted therapies, like sorafenib [64]. In fact, clinical trials testing unselected patients did not report a significant efficacy of the drug [42, 43]. Thus, we propose that the tumor dependence on bFGF and the high efficacy of sorafenib in inhibiting the FGFR axis might help with predicting tumor responsiveness to sorafenib.

## Conclusions

We report that sorafenib is a powerful inhibitor of MPM TIC proliferation and survival, mainly acting on FGFR1, whose level of activation directly correlates with drug efficacy and potency (Fig. 9). This distinctive mechanism may allow the selection of patient subsets in whom autocrine bFGF activation loops are active and who are more likely to better respond to sorafenib monotherapy or to association with drugs targeting alternative/converging Raf-dependent and independent intracellular pathways.

## Additional file

Additional file 1: Table S1. Analysis of the expression of surface markers (% of positive cells) and tumorigenicity of human MPM primary cultures. (DOCX 13 kb)

## Abbreviations

bFGF: Basic fibroblast growth factor
CSC: Cancer stem cell
EGF: Epidermal growth factor
MPM: Malignant pleural mesothelioma
TIC: Tumor-initiating cell
TKI: Tyrosine kinase inhibitor
