# Supplementary material for: The inhibition of FGF receptor 1 activity mediates sorafenib antiproliferative effects in human malignant pleural mesothelioma tumor-initiating cells
Source: Stem Cell Res Ther. 2017 May 25;8:119. doi: 10.1186/s13287-017-0573-7 (PMC5445511; doi:10.1186/s13287-017-0573-7)
Supplement: Additional file 1: Table S1. — Analysis of the expression of surface markers (% of positive cells) and tumorigenicity of human MPM primary cultures. (DOCX 13 kb) [file 13287_2017_573_MOESM1_ESM.docx]

**Additional file 1: Table S1. Analysis of the expression of surface markers (% of positive cells) and tumorigenicity of human MPM primary cultures.**

| Tumor code # | CD11b | CD24 | CD26 | CD30 | CD31 | CD46 | CD47 | CD55 | CD56 | CD63 | CD68 | CD86 | CD90 | CD99 | CD  140b | CD  141 | CD  184 | EGFR | *In vitro*  growth | *In vivo*  growth |
| --- | --- | --- | --- | --- | --- | --- | --- | --- | --- | --- | --- | --- | --- | --- | --- | --- | --- | --- | --- | --- |
| MM1 | 12 | 6 | 78 | - | - | 99 | 99 | 99 | 80 | 83 | 0 | 28 | 99 | 86 | 62 | 39 | - | 99 | + | + |
| MM2 | 22 | 28 | 8 | 2 | - | 77 | 99 | 97 | 31 | 66 | - | - | 92 | 98 | - | - | - | 95 | + | + |
| MM3 | 45 | 45 | 32 | - | - | 82 | 98 | 97 | 48 | 64 | 14 | 18 | 77 | 92 | 26 | 25 | 2 | 100 | + | + |
| MM4 | 0 | 46 | 92 | - | - | 87 | 99 | 98 | 43 | 50 | - | - | 72 | 98 | 17 | 18 | - | 100 | + | + |
| MM5 | 22 | 16 | 81 | - | 1 | 77 | 92 | 96 | 71 | 48 | - | 9 | 66 | 97 | 64 | 35 | - | 100 | + | - |
| MM6 | 21 | 10 | 78 | 2 | - | 73 | 99 | 100 | 47 | 37 | - | 12 | 85 | 91 | 4 | 12 | - | 97 | + | - |
| MM7 | 18 | 8 | 76 | 3 | 4 | 77 | 98 | 99 | 23 | 36 | - | 6 | 75 | 74 | 56 | 18 | - | 100 | + | - |
| MM8 | 32 | 10 | 51 | 4 | - | 71 | 99 | 100 | 68 | 41 | - | 10 | 97 | 88 | 12 | 2 | - | 99 | + | - |
| MM9 | 9 | 4 | 6 | - | - | 94 | 99 | 96 | 57 | 86 | - | 4 | 85 | 85 | 32 | 44 |  | 99 | + | - |
| MM10 | 6 | 15 | 88 | - | - | 85 | 99 | 99 | 68 | 77 | - | 30 | 81 | 89 | - | - | 5 | 100 | + | - |
